# *In Vitro* and *In Vivo* Effect of Xylopic Acid on Cytochrome P450 Enzymes

**DOI:** 10.1155/2022/4524877

**Published:** 2022-01-21

**Authors:** Mary A. Agbenyeku, Regina Appiah-Opong, Ernest Obese, Robert P. Biney, Emmanuel A. Adakudugu, Arnold D. Forkuo, Silas A. Osei, Mustapha K. Abeka, Elvis O. Ameyaw

**Affiliations:** ^1^Noguchi Memorial Institute for Medical Research, Department of Clinical Pathology, University of Legon, Accra, Ghana; ^2^Department of Biomedical Science, School of Allied Health Sciences, College of Health and Allied Sciences, University of Cape Coast, Cape Coast, Ghana; ^3^School of Pharmacy and Pharmaceutical Sciences, College of Health and Allied Sciences, University of Cape Coast, Ghana; ^4^Faculty of Pharmacy and Pharmaceutical Sciences, College of Health Sciences, Kwame Nkrumah University of Science and Technology, Kumasi, Ghana

## Abstract

**Introduction:**

Xylopic acid (XA), the major constituent of the fruit of *Xylopia aethiopica*, has shown several pharmacological properties. Traditionally, the plant is used to treat several diseases and is being used in the preparation of several local foods despite the lack of information about its safety, food-drug interaction, and other pharmacokinetic properties. This study, therefore, investigated the effect of XA on rat liver cytochrome P450 (CYP) enzymes *in vivo* and *in vitro*.

**Methods:**

Inhibition or induction of some isoforms of CYP450 enzymes: CYP 1A1/1A2, 1A2, 2B1/2B2, 3A4, 2D6, and 2C9 were investigated using microsomal fractions of the liver obtained from rats pretreated with a low dose of xylopic acid (LDT) 30 mg/kg, high dose of xylopic acid (HDT) 100 mg/kg, phenobarbitone (PC) 80 mg/kg, and ketoconazole (NC) 100 mg/kg, and a no-treatment group received distilled water, with (*n* = 5) animals in each group. The *in vitro* inhibition of CYP 3A4 was assessed by treating rat liver microsomes with XA.

**Results:**

Xylopic acid induced CYP 1A1/1A2, 1A2, 2D6, and 2C9, inhibited CYP 3A4, and had no effect on 2B1/2B2.

**Conclusion:**

The findings would help mitigate toxicity and therapeutic failure especially in cases of coadministration of medications with food containing XA, with metabolism altered by the latter.

## 1. Introduction

The use of medicinal natural products is traced back thousands of years due to the belief that herbal drugs have fewer side effects, enhance the effects of conventional agents, and can be an alternative for the management of several disorders [1, 2]. An example of one of such herbs is the *Xylopia aethiopica*. Studies have shown that the plant has anti-inflammatory, antimicrobial, analgesic, antiparasitic, and antioxidant properties [3–5]. In Ghana, the fruit is used by traditionalists to manage a wide range of disorders as it is believed to have curative effects on dyspepsia, cough, pain, and parasitic infections. It is equally used as a spice in the preparation of dishes such as porridge (Hausa Koko), sobolo, other African cuisines, and soups [5, 6]. Some food industries use it as a flavoring agent, and in some cosmetic industries, it is used as an agent for fragrance [7]. The plant is widely distributed in the West African rainforest from Senegal to Sudan in Eastern Africa and down to Angola in Southern Africa [8, 9] where it is mostly used for local cooking, especially in the preparation of what is referred to as the African pepper soup [10]. The plant grows prominently in several parts of Africa including Ghana, Nigeria, Benin, Kenya, Ivory Coast, and several other places [11]. Several studies have been performed on the fruit, and these studies reveal the plant contains about 98 compounds [12, 13]. Constituents of the fruit include essential oils, volatile oils, resin, arocene, rutheroside fat, bitter principles, alkaloids, glycosides, saponins, tannins, mucilage, xylopic acid, and kaurenoic acid [14]. The compounds obtained from the fruit that are considered most relevant are xylopic acid and kaurenoic acid. Xylopic acid, a kaurene diterpene, occurs as the major constituent in the fruits of *Xylopia aethiopica* [15].

**Figure 1:**
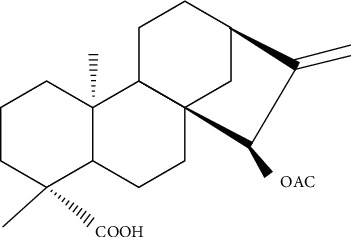
Chemical structure of xylopic acid [5].

Research on xylopic acid has shown that it exhibits diuretic and vasorelaxant properties in rodents [16]. Xylopic acid caused a substantial reduction of lung inflammation induced by carrageenan in mice, which supports the traditional use of the plant extracts of *Xylopia aethiopica* as therapeutic agents in conditions associated with acute inflammation and some respiratory disorders [17]. Xylopic acid possesses curative and prophylactic properties on *P. berghei*-induced malaria in ICR mice as well as antipyretic properties [18]. Xylopic acid and the fruit extract of *Xylopia aethiopica* were found to have significant central nervous system depressant effects in mice [3, 19]. The chemical structure of xylopic acid is shown in Figure 1.

Generally, people use traditional medicine with the notion that it has little or no side effects. Hence, such herbs do not pass through rigorous tests before approval is granted compared to conventional drugs [20, 21]. Due to insufficient scrutiny, most herbal medicines may result in CYP-mediated herb-drug interactions when coadministered with other orthodox medications, and this may go unnoticed [22]. Interactions of traditional medicines with human CYPs have been associated with alterations in the pharmacokinetics of several drugs [23]. They interact by either inhibiting or inducing CYPs which may result in harmful side effects. Drug-drug and herb-drug interactions is an important phenomenon that should be investigated before new agents are introduced on the market. Hence, it is useful to conduct series of investigations on the effect of traditional medicines on liver CYP450 drug-metabolizing enzymes [20]. This study sought to evaluate the effect of xylopic acid on carefully selected CYPs that form the major drug-metabolizing enzymes in the human system.

## 2. Materials and Methods

### 2.1. Drugs and Chemicals

Phenobarbitone, ketoconazole, CYP450 isoenzymes, potassium phosphate dibasic, potassium phosphate monobasic, bovine serum albumin (BSA), nicotinamide adenine dinucleotide phosphate (NADPH), ethoxyresorufin, methoxyresorufin, pentoxyresorufin, benzyloxyresorufin, dextromethorphan, diclofenac, zinc sulphate heptahydrate, trimethylamine, and acetonitrile used to evaluate the effect of xylopic acid on CYPs were all obtained from Sigma-Aldrich Chemie GmbH (Eschenstrasse), Germany. XA was purified following the procedure described by Biney et al. [24].

### 2.2. Experimental Animals

Before the experiment, male Sprague–Dawley rats were purchased from the Animal Experimentation Unit of Noguchi Memorial Institute for Medical Research, Accra, Ghana and were fed *ad libitum* using standard animal laboratory pellet and water. They were housed under standard laboratory conditions of 25 ± 1°C ambient temperature, 60–70% relative humidity, and 12 : 12 hour light : dark cycle to ensure acclimatization to the laboratory. The experiments were conducted following the National Institute of Health (NIH) Guidelines for Care and Use of Laboratory Animals and approved by the Scientific and Technical Committee and Institutional Animal Care and Use Committee (IACUC) of Noguchi Memorial Institute for Medical Research, University of Ghana (UG-IACUC004/18–19).

### 2.3. *In Vivo* CYP450 Isoenzyme Induction Assay

The activities of xylopic acid high dose (100 mg/kg) and xylopic acid low dose (30 mg/kg) were tested on CYP 1A1/1A2 (EROD), CYP 2B1/2B2 (PROD), CYP 3A4 (BROD), CYP 2D6, and CYP 2C9 employing *in vivo* and *in vitro* assays as earlier described [25]. Ketoconazole, sulfaphenazole, diclofenac, dextromethorphan, and phenobarbitone were used as standard drugs in the study.

Male rats weighing 250–300 g (*n* = 5) were randomly assigned to one of the four experimental groups, being low-dose xylopic acid treatment group (LDT) 30 mg/kg, high-dose xylopic acid treatment group (HDT) 100 mg/kg, phenobarbitone (PC) 80 mg/kg, a no-treatment group that received distilled water, and finally for CYP 3A4, a control group treated with ketoconazole (KC) 100 mg/kg with (*n* = 5) animals in each group. The choice of dosage for XA was informed by previous studies on the interaction of XA on hepatic enzymes using pentobarbitone-induced sleep test in mice [24]. The treatment for each group was administered orally for 7 days. All the animals were anesthetized by CO_2_ inhalation to minimize suffering, then sacrificed by abdominal venesection method after which their liver was harvested, snap-frozen in liquid nitrogen, and stored at −80°C until further analysis was carried out.

### 2.4. Preparation of Microsomal, Cytosolic Fractions

Liver samples of 7.5 g were obtained from each animal and kept in the −80°C freezer until use. On the day of the experiment, each liver sample was then homogenized in two volumes of 20 mM potassium phosphate buffer (pH 7.4) using a mortar and a pestle. The homogenate was centrifuged at 2,500 rpm for 50 minutes at 4°C and further centrifuged at 40,000 rpm for 60 minutes at 4°C. The resultant supernatant (cytosol) was then separated from the pellet (microsomes). The microsomes obtained were further homogenized in potassium phosphate buffer 20 mM (pH 7.4) and stored at −80°C [25, 26].

### 2.5. Protein Determination and Standardization

The protocol for protein determination was adapted from the protocol described by Harwood et al. [25, 26]. Fourfold serial dilutions were performed on the microsomal solutions using potassium phosphate buffer. Then, a twofold serial dilution for seven concentrations was prepared for a protein standard, bovine serum albumin (BSA). A volume of 10 *μ*L of BSA and 200 *μ*L of Bio-Rad reagent which was then added to each microsomal dilution in a 96-well plate in triplicate and incubated at room temperature for 5 minutes, after which the absorbance was read at a wavelength of 530 nm excitation and 586 nm emission using Tecan Infinite M200 PRO.

### 2.6. The Effect of XA on CYP 1A1/1A2, 1A2, 3A4, and 2B1/2B2 Enzymes

The effect of xylopic acid on CYP 1A1/1A2, 1A2, 2B1/2B2, and 3A4 was determined using fluorometric assays as described previously [19]. This was achieved by pipetting 70 *μ*L of potassium phosphate buffer at a pH of 7.4 in triplicate into a 96-well plate followed by addition of 10 *μ*L (2 *μ*M) of each substrate (ethoxyresorufin (EROD), methoxyresorufin (MROD), pentoxyresorufin (PROD), and benzyloxyresorufin (BROD)). Subsequently, 10 *μ*L of the microsomal fraction obtained from each treatment group was added to each well plate and preincubated at 37°C for 5 minutes, after which, 10 *μ*L of nicotinamide adenine dinucleotide phosphate (NADPH) with a concentration of 100 *μ*M was added to each of the wells and incubated at 37°C for 10, 20, and 30 minutes for CYPs 1A1/1A2, 1A2, 2B1/2B2, and 3A4, respectively. Plates were gently shaken after the addition of 40 *μ*L of stopping solution (20% 0.5 M Tris and 80% acetonitrile) to each well. Fluorescence was determined at wavelengths of 586 nm emission and 530 nm excitation proceeding optimization of the protocol. The assay was repeated, and the mean values were used for computation.

### 2.7. *In vitro* Inhibitory Activity of XA on CYP 3A4

Microsomes obtained from male rats pretreated with phenobarbitone were used in this assay. The standard inhibitor used was ketoconazole for CYP 3A4 and xylopic acid as the test compound. A cocktail was prepared for seven different concentrations for each standard inhibitor. The cocktail contained 3000 *μ*l of 0.1 M potassium phosphate buffer (pH 7.4) which was pipetted into a 50 ml Eppendorf tube, followed by the addition of 600 *μ*l of the substrate (benzyloxyresorufin) for CYP 3A4, and followed by the addition of 600 *μ*l of the enzyme (microsomes). A volume of 80 *μ*l was then pipetted from the cocktail into a 96-well plate in triplicate and then preincubated at 37°C for 5 minutes. Afterward, 10 *μ*L of nicotinamide adenine dinucleotide phosphate (NADPH) with a concentration of (100 *μ*M) was added to each of the wells followed by the addition of ketoconazole and xylopic acid to separate plates containing BROD. The plates were incubated at 37°C for 30 minutes for BROD. A volume of 40 *μ*L of stopping solution (20% 0.5 M Tris and 80% acetonitrile) was finally added to each well. The plate was gently shaken, and its fluorescence read at wavelengths of 586 nm emission and 530 nm excitation [19]. The assay was repeated, and the mean values were used for computation.

### 2.8. Effect of XA on CYP 2D6

The CYP 2D6-dextromethorphan-O-demethylation assay was used to determine the effect of xylopic acid on CYP 2D6. The assay was done by pipetting 350 *μ*L of potassium phosphate buffer (pH 7.4) into Eppendorf tubes in triplicate. A volume of 50 *μ*L of 0.25 mM dextromethorphan was added, followed by 50 *μ*L of microsomes obtained from each treatment group. The mixture was preincubated at 37°C for 5 minutes in a water bath after which 50 *μ*L of NADPH solution 100 *μ*M was added. The mixture was further incubated for 45 minutes, followed by the addition of 100 *μ*L of stopping solution (300 mM zinc sulphate heptahydrate). The mixture was centrifuged at 4,000 rpm for 15 min at room temperature, and the supernatant was collected in vials. Analysis of the supernatant was done using an isocratic HPLC (Shimadzu Prominence, Japan). The mobile phase consisted of 24% (v/v) acetonitrile and 0.1% (v/v) trimethylamine adjusted to pH 3.0 with perchloric acid. The carrier flow rate was 0.8 ml/min, and peaks were monitored at wavelengths of 280 nm excitation and 310 nm emission [19]. The assay was repeated, and the mean values were used for computation.

### 2.9. Effect of XA on CYP 2C9

The CYP 2C9 diclofenac hydroxylation assay was used to determine the effect of xylopic acid on CYP 2C9 [26]. This assay measures the effect of XA on hydroxylation of diclofenac to 4-hydroxydiclofenac by CYP 2C9. This was done by pipetting 350 *μ*L of potassium phosphate buffer (pH 7.4) into Eppendorf tubes followed by adding 50 *μ*L of 0.05 mM diclofenac, after which 50 *μ*L of the microsomal fraction obtained from each treatment group was added (in triplicate) and preincubated at 37°C for 5 minutes in a water bath. A volume of 50 *μ*L of NADPH solution (100 *μ*M) was added to each tube and further incubated in a water bath at 37°C for 10 minutes. The reaction was terminated by adding 200 *μ*L of stopping solution (ice-cold methanol) to the mixture. The mixture was centrifuged at 12,000 rpm for 8 minutes at room temperature. The supernatants were collected in vials and analyzed using high-performance liquid chromatography [15]. The assay was repeated, and the mean values were used for computation.

### 2.10. Data Analysis

For the *in vivo* testing, GraphPad Prism version 8 was used for all statistical analyses, and *P* values < 0.05 were considered statistically significant. All data obtained were expressed as a mean ± standard error of the mean. For the *in vitro* analysis, the column graphs were subjected to one-way ANOVA with Tukey's post *hoc* test. Doses for 50% of the maximal effect (IC_50_) for each drug were determined by using an iterative computer least squares method, with the following nonlinear regression (3-parameter logistic) equation. The fitted midpoints (IC_50_s) of the curves were compared statistically using the F test.

## 3. Results

### 3.1. Effect of Phenobarbitone and Xylopic Acid High and Low Dose on CYP 1A2

**Figure 2:**
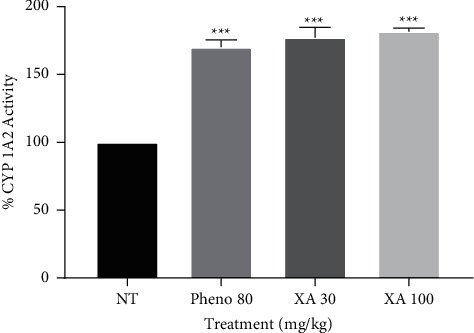
Effect of phenobarbitone (Pheno 80), xylopic acid low dose (30 mg/kg; XA 30), and xylopic acid high dose (100 mg/kg; XA 100) on CYP 1A2 (MROD) enzyme activity. Data are presented as mean ± S.E.M. ^∗∗∗^*P* < 0.001 compared to no-treatment group (one-way ANOVA followed by Tukey's post *hoc* test).

All the treatment groups produced significant (*P* < 0.05) CYP 1A2 enzyme induction as shown in Figure 2. Administration of xylopic acid at a high dose of 100 mg/kg induced CYP 1A2 by 81.60% while low dose of 30 mg/kg by 77.20%. Phenobarbitone pretreatment induced CYP 1A2 by 70.10% as compared to the nontreatment group that served as the control.

### 3.2. Effect of Phenobarbitone and Xylopic Acid on CYP 1A1/1A2

**Figure 3:**
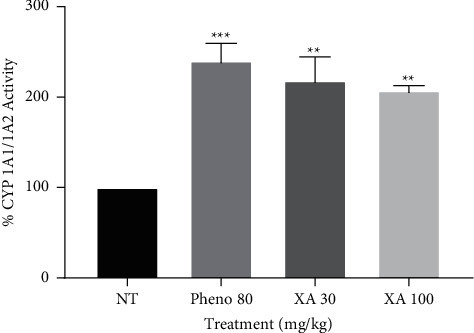
Effect of phenobarbitone (Pheno 80), xylopic acid low dose (30 mg/kg; XA 30), and xylopic acid high dose (100 mg/kg; XA 100) on CYP 1A1/1A2 (EROD) enzyme activity. Data are presented as mean ± S.E.M. *P* < 0.01, ^∗∗∗^*P* < 0.001 compared to the negative control (no treatment) group (one-way ANOVA followed by Tukey's post *hoc* test).

All the treatment groups had a significant (*P* < 0.05) effect on CYP 1A1/1A2, with phenobarbitone alone exhibiting the highest enzyme induction with a percentage activity of 139.9% (Figure 3). A high dose of xylopic acid (100 mg/kg) induced the enzyme resulting in an activity of 106.9% while xylopic acid low dose induced the enzyme with an activity of 118% compared to the negative control. The efficacy order for the various treatment groups on CYP 1A1/1A2 was phenobarbitone > xylopic acid low dose > xylopic acid high dose.

### 3.3. Effect of Phenobarbitone and Xylopic Acid on CYP 2B1/2B2

**Figure 4:**
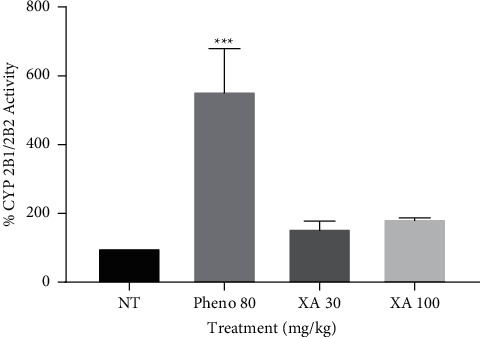
Effect of phenobarbitone (Pheno 80), xylopic acid low dose (30 mg/kg; XA 30), and xylopic acid high dose (100 mg/kg; XA 100) on CYP 2B1/2B2 enzyme. Data are presented as mean ± S.E.M. ^∗∗∗^*P* < 0.001 compared to the no-treatment group (one-way ANOVA followed by Tukey's post *hoc* test).

From Figure 4, the phenobarbitone pretreatment group had the most significant (*P* < 0.001) effect on CYP 2B1/2B2. XA low and high doses did not produce a significant effect on CYP 2B1/2B2 compared to the no treatment group. However, there was about a 2.5-fold increase in CYP 2B1/2B2 activity of phenobarbitone-treated animals compared to both doses of XA.

### 3.4. Effect of Phenobarbitone and Xylopic Acid on CYP 3A4

**Figure 5:**
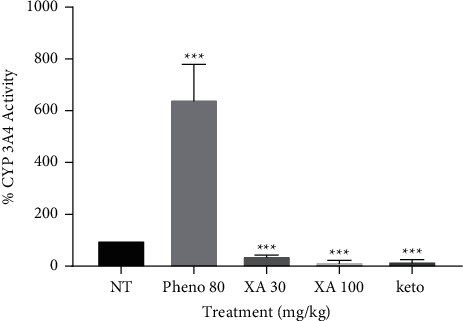
Effect of phenobarbitone (Pheno 80), xylopic acid low dose (30 mg/kg; XA 30), and xylopic acid high dose (100 mg/kg; XA 100), and ketoconazole (keto) on CYP 3A4 enzyme. Data are presented as mean ± S.E.M ^∗∗∗^*P* < 0.001 compared to the no-treatment group (one-way ANOVA followed by Tukey's post *hoc* test).

Figure 5 illustrates the activity of phenobarbitone, ketoconazole, and XA low and high doses. Phenobarbitone induced CYP 3A4 with an about 6-fold increase in activity, while ketoconazole and XA low and high dose produced about 2- to 3-fold inhibition of CYP 3A4 activities. Comparatively, the inhibitory activity of XA high dose was approximately 1.36 times more than the standard ketoconazole.

### 3.5. Effect of Phenobarbitone and Xylopic Acid on CYP 2C9

**Figure 6:**
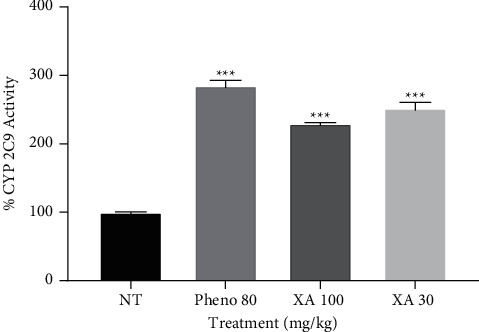
Effect of phenobarbitone (Pheno 80), xylopic acid low dose (30 mg/kg; XA 30), and xylopic acid high dose (100 mg/kg; XA 100) on CYP 2C9 (diclofenac hydroxylation). Data are presented as mean ± S.E.M ^∗∗∗^*P* < 0.001 compared to the no-treatment group (one-way ANOVA followed by Tukey's post *hoc* test).

Figure 6 shows the effect of phenobarbitone and xylopic acid low and high dose on the biotransformation of diclofenac by CYP 2C9. All the treatment groups significantly induced the enzyme activity with percentage enzyme induction of 184.9%, 129.9%, and 151.8% for phenobarbitone, xylopic acid low dose, and xylopic acid high dose, respectively, as compared to the no-treatment group (*P* < 0.001).

### 3.6. Effect of Phenobarbitone and Xylopic Acid on CYP 2D6

**Figure 7:**
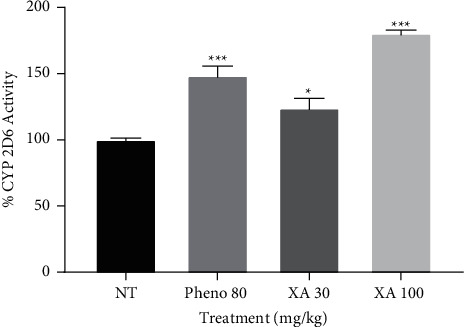
Effect of phenobarbitone (Pheno 80), xylopic acid low dose (30 mg/kg; XA 30), and xylopic acid high dose (100 mg/kg; XA 100) on CYP 2D6 (dextromethorphan O-demethylation). Data are presented as mean ± S.E.M. ^*∗*^*P* < 0.05 and ^∗∗∗^*P* < 0.001 compared to the no-treatment group (one-way ANOVA followed by Tukey's post *hoc* test).

Effects of phenobarbitone, XA low dose, and XA high dose on CYP 2D6 are shown in Figure 7. The figure describes a significance in the difference between CYP 2D6 activity of the negative control experiment and the treated (*P* < 0.001) for phenobarbitone and XA high dose and a significance of (*P* < 0.05) in the case of XA low dose. Phenobarbitone, XA low dose, and XA high dose had activities of 48.6%, 24%, and 80.5%, respectively. XA high dose was 1.65 times more effective (*P*=0.0029) than the standard phenobarbitone.

### 3.7. Effect of Xylopic Acid on CYP 3A4 *In Vitro*

**Figure 8:**
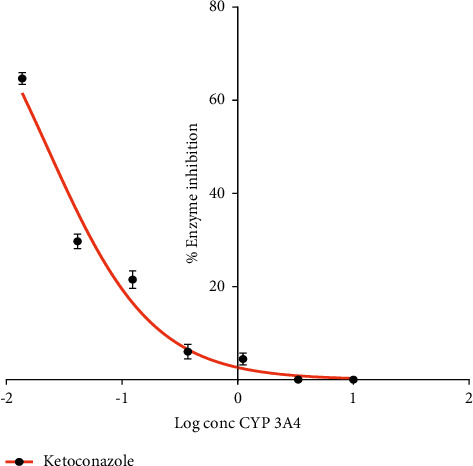
Dose-response curve of ketoconazole on the inhibition of CYP 3A4. Ketoconazole was used as the standard drug and various concentrations were used to determine the IC_50_ values. Data are presented as mean ± S.E.M.

**Figure 9:**
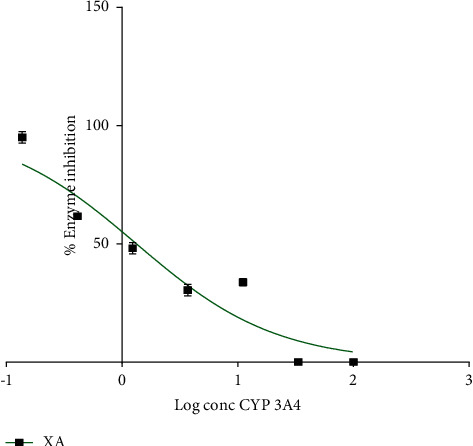
Dose-response curve of varying concentrations of XA against enzyme inhibition on CYP 3A4. XA was added at various concentrations to determine the IC_50_ value. Data are presented as mean ± S.E.M. Nonlinear regression analysis was used in plotting the curve.

Figures 8 and 9 show the effect of various concentrations of ketoconazole and xylopic acid on CYP 3A4 *in vitro*. Ketoconazole was used as the positive control. The IC_50_ values for ketoconazole and xylopic acid were 0.091 ± 0.002 *μ*M and 1.30 ± 0.01 *μ*M, respectively. Thus, ketoconazole showed more potent inhibition of CYP 3A4 than xylopic acid.

## 4. Discussion

The study evaluated the ability of xylopic acid to influence the activity of hepatic CYPs in rats *in vitro* and *in vivo*. Interestingly, a recent study suggested that xylopic acid has a biphasic effect on rat hepatic CYP450 enzymes at low and high doses [24], but no specific enzymes were implicated in the biotransformation of the compound. Again, there are no studies on the effect of xylopic acid or the fruit of *X. aethiopica* (which contains xylopic acid) on human hepatic CYP450 enzymes although the fruits are heavily consumed as a spice in local dishes. Therefore, there are no data on the metabolism of xylopic acid or its related kaurene diterpenes in rodents or humans, hence limiting these studies to implicate any possible metabolite of xylopic acid on the effects observed in these studies. Owing to the fact that *X. aethiopica* is heavily consumed, xylopic acid or its possible active metabolites can reach concentrations high enough to interact with other medications when taken concurrently. It was therefore imperative to identify the specific isoenzymes induced and inhibited on the administration of xylopic acid with a focus on CYP 1A1/1A2, 1A2, 2B1/2B2, 3A4, 2D6, and 2C9.

Xylopic acid induced CYP 1A1/1A2 at high and low doses. Other substances that induce CYP 1A1/1A2 are barbiturates, tobacco, and rifampin among others [27].

Xylopic acid induced CYP 1A2 *in vivo* at low and high doses, like the effect seen in phenobarbitone. Studies have shown that CYP 1A2 is responsible for the primary metabolism of theophylline and propranolol amongst others [28]. A study implicated cimetidine which is a histamine H_2_-receptor antagonist as an inhibitor of CYP 1A2 [28]. This suggested that the administration of xylopic acid with H_2_-receptor antagonists may result in drug-drug interaction.

CYP 2B1/2B2 is a phenobarbital-inducible member of the P450 xenobiotic-inducible superfamily involved in the hydroxylation of decanoic and other fatty acids [29]. The current study revealed that xylopic acid had no activity (induction/inhibition) on CYP 2B1/2B2.

CYP 3A4 was inhibited by low and high doses of xylopic acid. The inhibition observed was weaker than that expressed by ketoconazole, a standard inhibitor of 3A4. CYP 3A4 accounts for 30–50% of drugs metabolized through type I enzymes [30]; therefore, it was paramount to investigate the effect of xylopic acid on CYP 3A4. Although the dose of xylopic acid used in the *in vivo* studies were 30 and 100 mg/kg, the amount absorbed into the bloodstream was not measured, but the concentration achieved in the blood can be high enough to produce food-drug interaction since this concentration was able to inhibit the CYP 3A4 enzymes.

Xylopic acid at low and high doses induced CYP 2C9. It is primarily expressed in the liver; a study suggests that its expression level is the second highest among CYP isoforms. It is responsible for the metabolic clearance of up to 15–20% of all drugs [31]. Some other substrates metabolized by this CYP aside from diclofenac include alkylating agents such as cyclophosphamide [32] and antiestrogenic drugs such as tamoxifen [33]. Treatment with rifampicin has been implicated in increased clearance of drugs metabolized by CYP 2C9 [32]. Drugs that inhibit this enzyme include fluconazole and sulfaphenazole. There is evidence supporting the danger of drug-drug interaction when sulfaphenazole is added to a therapeutic regime that includes drugs with a low therapeutic index, such as warfarin [34]. This suggests that the use of xylopic acid with these inhibitors should be carefully considered.

Patients vary widely in their response to drugs. They include individuals with no CYP 2D6 activity and individuals with genetically elevated CYP 2D6 activity, suggesting an altered response to drugs that are CYP 2D6 substrates. Drugs that inhibit CYP 2D6 activity are likely to increase the plasma concentrations of other medications, such as fluoxetine and paroxetine leading to drug toxicity [35].

## 5. Conclusion

Xylopic acid induced rat liver microsomal CYP 1A1/1A2, 1A2, 2D6, and 2C9; inhibited CYP 3A4; and did not affect CYP 2B1/2B2. Since these enzymes are implicated in the metabolism of several drugs, it is expected that XA can potentially influence the metabolism of other drugs through food-drug and drug-drug interactions.

## Data Availability

The data in support of the findings of this study may be requested and obtained from the corresponding author.
